# Identification of Ferroptosis-Related Hub Genes Linked to Suppressed Sulfur Metabolism and Immune Remodeling in *Schistosoma japonicum*-Induced Liver Fibrosis

**DOI:** 10.3390/pathogens15020126

**Published:** 2026-01-23

**Authors:** Yin Xu, Hui Xu, Dequan Ying, Jun Wu, Yusong Wen, Tingting Qiu, Sheng Ding, Yifeng Li, Shuying Xie

**Affiliations:** 1 Jiangxi Provincial Institute of Parasitic Diseases, Nanchang 330096, China; jxjysxuyin@163.com (Y.X.); xh15279497377@163.com (H.X.); wlmtxm@163.com (J.W.); 15907087560@163.com (Y.W.); jxncqtt2017@163.com (T.Q.); jxcdccfs2@126.com (S.D.); 2 School of Medical Imaging, Nanchang Medical College, Nanchang 330052, China; yingdq@outlook.com

**Keywords:** *Schistosoma japonicum*, liver fibrosis, ferroptosis, sulfur metabolism, immune microenvironment, hub genes

## Abstract

Liver fibrosis induced by *Schistosoma japonicum* Katsurada, 1904 (*S. japonicum*) infection lacks effective diagnostic markers and specific anti-fibrotic therapies. Although dysregulated iron homeostasis and ferroptosis pathways may contribute to its pathogenesis, the core regulatory mechanisms remain elusive. To unravel the ferroptosis-related molecular features, this study integrated transcriptomic datasets (GSE25713 and GSE59276) from *S. japonicum*-infected mouse livers. Following batch effect correction and normalization, ferroptosis-related differentially expressed genes (FRDEGs) were identified. Subsequently, core hub genes were screened through the construction of a protein–protein interaction (PPI) network, functional enrichment analysis, immune infiltration evaluation, and receiver operating characteristic (ROC) analysis. The expression patterns of these hub genes were further validated in an *S. japonicum*-infected mouse model using reverse transcription-quantitative polymerase chain reaction (RT-qPCR). The study identified 7 hub genes (*Lcn2*, *Timp1*, *Cth*, *Cp*, *Hmox1*, *Cbs*, and *Gclc*) as key regulatory molecules. Functional enrichment analysis revealed that these hub genes are closely associated with sulfur amino acid metabolism and oxidative stress responses. Specifically, key enzymes involved in cysteine and glutathione (GSH) synthesis (*Cth*, *Cbs*, *Gclc*) were consistently downregulated, suggesting a severe impairment of the host antioxidant defense capacity. Conversely, pro-fibrotic and pro-inflammatory markers (*Timp1*, *Lcn2*, *Hmox1*) were upregulated. This molecular pattern was significantly associated with a remodeled immune microenvironment, characterized by increased infiltration of neutrophils and eosinophils. In vivo validation confirmed the expression trends of 6 hub genes, corroborating the bioinformatics predictions, while the discrepancy in *Cp* expression highlighted the complexity of post-transcriptional regulation in vivo. The identified hub genes demonstrated excellent diagnostic potential, with *Timp1* achieving an area under the curve (AUC) of 1.000. This study elucidates the molecular link between *S. japonicum* infection and the ferroptosis pathway, suggesting that these hub genes may drive liver fibrosis progression by regulating sulfur metabolism and the immune microenvironment. These findings offer potential diagnostic biomarkers and novel therapeutic targets for schistosomal liver fibrosis.

## 1. Introduction

Schistosomiasis is a globally prevalent tropical and subtropical zoonotic parasitic disease closely associated with poverty and inadequate sanitation. It is estimated that approximately 1 billion people worldwide are at risk of infection, with 250 million people across 78 countries affected by the disease [1]. The major *Schistosoma* species infecting humans include *Schistosoma japonicum* Katsurada, 1904 (*S. japonicum*), *Schistosoma mansoni* Sambon, 1907 (*S. mansoni*), and *Schistosoma haematobium* (Bilharz, 1852) *(S. haematobium*). Infection initiates when the host’s skin contacts cercariae released by freshwater snails. Following oviposition by adult worms, eggs become trapped in tissues such as the liver (mainly *S. japonicum* and *S. mansoni*) or bladder (*S. haematobium*). This induces the host immune system to release cytokines, triggering inflammation and granuloma formation, which ultimately leads to fibrosis [2].

Liver fibrosis induced by *S. japonicum* is primarily driven by egg deposition. Eggs trapped in the hepatic portal vein branches release a complex mixture of antigenic components known as soluble egg antigen (SEA). SEA drives a potent immune response, resulting in granuloma formation, immune cell infiltration, and hepatocyte injury. This process critically activates hepatic stellate cells (HSCs), leading to excessive collagen deposition, and ultimately progressing into irreversible pipestem fibrosis [3]. Therefore, deciphering the signaling networks underlying SEA-mediated pathogen–host interactions and identifying key molecular targets is of great significance for developing novel intervention strategies against schistosomal liver fibrosis.

The interaction between *S. japonicum* and host iron metabolism constitutes a core component of the pathogen–host relationship. The growth and development of this parasite, particularly the maturation and oviposition of female worms, are highly dependent on the acquisition of host iron resources [4]. Given the indispensable role of iron in egg formation, vaccines or therapeutic strategies targeting iron homeostasis hold promise for significantly alleviating egg-induced pathologies, including liver fibrosis [5]. Furthermore, studies have confirmed that iron chelators can reduce hepatic egg burden and inhibit HSC activation by depriving schistosomes of essential iron, ultimately ameliorating liver fibrosis [6]. However, the mechanism of iron deprivation alone is insufficient to fully explain the complex pathological process. Whether the imbalance of host iron metabolism drives the development of liver fibrosis by inducing specific types of hepatocyte death remains to be elucidated. Ferroptosis, a form of regulated cell death driven by iron-dependent lipid peroxidation, provides a key entry point for deciphering this mechanism.

Ferroptosis is an iron-dependent form of programmed cell death characterized by the accumulation of reactive oxygen species (ROS), depletion of glutathione (GSH), and elevated levels of lipid peroxidation products. Sulfur metabolic pathways, particularly cysteine and GSH synthesis, serve as central regulatory mechanisms of ferroptosis [7]. Accumulating evidence suggests that ferroptosis is a critical regulatory pathway in the progression of liver fibrosis. Ferroptosis in hepatocytes can induce the release of pro-inflammatory factors and damage-associated molecular patterns (DAMPs), which subsequently activate HSCs and promote collagen deposition, thereby exacerbating the fibrotic process [8,9]. Studies have shown that parasitic infections can disrupt host cell iron homeostasis by sequestering host iron, upregulating ROS production, or downregulating ferroptosis inhibitors [10,11]. Although *S. japonicum* actively consumes host iron, complex metabolic disturbances in the hepatic microenvironment, particularly around egg granulomas, can lead to local iron overload and lipid peroxidation; notably, iron accumulation is the core initiating condition for ferroptosis [12]. Nevertheless, the specific mechanisms by which *S. japonicum* and SEA interact with local iron homeostasis and the ferroptosis pathway to drive fibrosis, as well as the core genes regulating this process, remain largely unclear.

To address these gaps, this study integrated public gene expression datasets (GSE25713 and GSE59276) and employed bioinformatics approaches, including differential expression analysis, functional enrichment, and protein–protein interaction (PPI) network construction, alongside reverse transcription-quantitative polymerase chain reaction (RT-qPCR) validation in an *S. japonicum*-infected mouse model. We aimed to identify hub regulatory genes associated with ferroptosis in schistosomal liver fibrosis and elucidate their potential molecular mechanisms. By systematically characterizing the molecular targets linking pathogen–host interactions to the ferroptosis pathway, this study offers a novel perspective on the synergistic effects of parasite-induced iron homeostasis imbalance and cell death in liver fibrosis. Furthermore, our findings provide theoretical support and candidate biomarkers with potential translational value for addressing current clinical challenges related to diagnostic markers and antifibrotic therapies.

## 2. Materials and Methods

### 2.1. Data Download

Two mouse liver gene expression datasets related to *S. japonicum* infection, GSE25713 [13] and GSE59276, were retrieved from the Gene Expression Omnibus (GEO) database [14] using the GEOquery package (Version 2.76.0) [15]. The GSE25713 dataset, based on the GPL6887 platform, comprised 24 mouse liver samples, including 18 *S. japonicum*-infected samples and 6 controls. The GSE59276 dataset was also derived from mouse liver tissue using the GPL6885 data platform. The original submission to the GEO database included 36 samples. After excluding 22 praziquantel-treated samples from the original 36 submissions, 14 samples without intervention were retained for this study: 12 *S. japonicum*-infected samples and 2 controls. Detailed information on the datasets is provided in Supplementary Table S1.

Ferroptosis-related genes (FRGs) were retrieved from the GeneCards [16] (https://www.genecards.org/, accessed on 4 June 2024) and Molecular Signatures Database (MSigDB) (http://www.gsea-msigdb.org/gsea/msigdb/human/search.jsp, accessed on 4 June 2024) databases. The GeneCards database provides detailed information on human genes, which can be converted to mouse genes through gene IDs. FRGs were identified in the GeneCards database using the keyword “ferroptosis”. Screening criteria were set to “protein-coding” with a “relevance score” > 2, resulting in the identification of 92 FRGs. In addition, 67 FRGs were obtained from the MSigDB database. FRGs were merged and duplicates were removed, resulting in 99 unique FRGs (Supplementary Table S2).

### 2.2. Analysis of Differentially Expressed Genes (DEGs)

To investigate the DEGs between the infected group and the control group, the GSE25713 and GSE59276 datasets were first merged based on common gene symbols. Subsequently, batch effect correction was performed using the sva (Version 3.56.0) and limma (Version 3.64.1) packages to eliminate technical biases and normalize the data. The limma package [17] was then used for differential expression analysis of the processed expression profile data. Genes meeting the criteria of |log fold change (logFC)| > 0.5 and adjusted *p*-value (*p*.adj) < 0.05 were designated as DEGs. Specifically, genes with a logFC > 0.5 and *p*.adj < 0.05 were classified as upregulated, while those with a logFC < −0.5 and *p*.adj < 0.05 were classified as downregulated.

Ferroptosis-related differentially expressed genes (FRDEGs) were identified by intersecting the merged DEGs (characterized by |logFC| > 0.5 and *p*.adj < 0.05) with FRGs retrieved from the GeneCards and MSigDB databases. A Venn diagram was used to visualize this intersection, and the resulting genes were designated as FRDEGs for further analysis. A volcano plot showing the results of differential analysis was generated using the R package ggplot2 (v3.4.4; https://ggplot2.tidyverse.org, accessed on 4 June 2024), while a heatmap of FRDEGs was generated using the R package pheatmap.

### 2.3. Gene Function and Pathway Enrichment Analysis

Gene Ontology (GO) enrichment analysis [18] was performed to annotate biological process (BP), molecular function (MF), and cellular component (CC) terms [19]. The Kyoto Encyclopedia of Genes and Genomes (KEGG) is a comprehensive database that provides information on genomes, biological pathways, diseases, and drugs [20]. GO and KEGG pathway enrichment analyses of DEGs were conducted using the R package clusterProfiler [21]. An entry was considered statistically significant if it met the criteria of *p* < 0.05 and false discovery rate (FDR) < 0.25.

### 2.4. Gene Set Enrichment Analysis (GSEA)

To evaluate the contribution of genes to the phenotype, GSEA was performed on the merged dataset sorted by logFC using the clusterProfiler package (Version 4.16.0) [22]. GSEA parameters were set as follows: random seed 2022, 5000 permutations, and gene set size restricted to a minimum of 10 and a maximum of 500 genes. The gene set “c2.cp.V2022.1.Mm.Symbols.gmt” (canonical pathways) for *Mus musculus* Linnaeus, 1758 was retrieved from MSigDB [23]. Significant enrichment was determined by Benjamini–Hochberg (BH)-corrected *p* values, with the criteria set as *p*.adj < 0.05 and FDR < 0.25.

### 2.5. Differential Expression and ROC Analysis of FRDEGs

We used the Wilcoxon rank-sum test to examine the expression differences in FRDEGs between the infected and control groups in the merged dataset, and visualized these differences using ggplot2. In addition, we performed diagnostic analysis by plotting receiver operating characteristic (ROC) curves of FRDEGs [24]. The R package pROC was used to generate ROC curves and calculate the area under the curve (AUC) values to evaluate the diagnostic performance of FRDEG expression for the disease, where an AUC value closer to 1 indicates superior diagnostic accuracy.

### 2.6. ImmuCC for Immune Infiltration Analysis

To evaluate the immune landscape of *S. japonicum*-infected mouse livers, we performed immune cell infiltration analysis using the ImmuCC method [25]. This computational tool leverages the CIBERSORT deconvolution framework based on linear support vector regression to estimate the composition and relative abundance of immune cells within mixed tissue samples. Given the murine origin of the transcriptomic data, ImmuCC employs a specifically optimized mouse-specific reference signature matrix (instead of the standard human LM22 reference) to quantify 25 distinct murine immune cell subtypes. To ensure the accuracy of the analysis, immune cell subtypes with an estimated relative abundance of zero across all samples were excluded. The resulting immune infiltration matrix was visualized using a stacked bar chart to display the proportional composition of immune cells. The Wilcoxon rank-sum test was used to assess differences in the proportions of the 25 immune cell types between the infected and control groups, and only cell subtypes showing statistically significant differences were retained for subsequent analysis.

To investigate interactions within the immune microenvironment, Spearman correlation analysis was performed among the differentially infiltrated immune cells, and the results were visualized as a heatmap using the R package pheatmap. Furthermore, Spearman correlation analysis was conducted to evaluate the associations between FRDEGs and infiltrating immune cells. Correlations with a *p* < 0.05 were considered statistically significant and were visualized as a bubble chart using the R package ggplot2.

### 2.7. PPI Network and Functional Similarity Analysis

To investigate protein–protein interactions, a PPI network was constructed for the 10 FRDEGs using the STRING database [26]. The organism was set to *Mus musculus*, and the minimum interaction score threshold was defined as high confidence (0.700). Genes that did not form interactions with other nodes in the network were excluded. The remaining 7 interconnected nodes formed the core regulatory network and were designated as hub genes. The resulting network was visualized using Cytoscape software (Version 3.9.1) [27].

Subsequently, the GOSemSim package (Version 2.34.0) [28] was utilized to evaluate the GO semantic similarity of the hub genes. The geometric mean was calculated across BP, CC, and MF categories to derive a final similarity score. The results of the functional similarity analysis were visualized using the R package ggplot2.

Furthermore, we integrated genomic and proteomic data using the GeneMANIA database [29] to predict candidate genes functionally similar to the hub genes. An interaction network was constructed via the GeneMANIA online platform, where the inner circle represents the queried hub genes and the outer circle represents the predicted functionally related genes. Lines between nodes indicate shared functional interactions.

### 2.8. Animal Model

Eight specific pathogen-free (SPF) male BALB/c mice (6–8 weeks old) were purchased from SPF (Beijing) Biotechnology Co., Ltd. (Beijing, China). These mice were housed at Jiangxi Zhonghong Boyuan Biotechnology Co., Ltd. (Nanchang, China), under standardized conditions with a temperature controlled between 20 °C and 26 °C and humidity maintained between 40% and 70%. Mice had free access to food and water and were kept on a 12-h light/dark cycle. *Oncomelania hupensis* Gredler, 1881 (*O. hupensis*) infected with *S. japonicum* was provided by the Jiangxi Institute of Parasitic Diseases, Nanchang, China. *O. hupensis* was maintained in Petri dishes containing moist filter paper.

The eight mice were randomly divided into a control group (*n* = 4) and an infected group (*n* = 4). *O. hupensis* was placed in chlorine-free water and exposed to incandescent light for 2 h to promote cercariae release. Mice in the infected group were anesthetized with an intraperitoneal injection of 0.6% sodium pentobarbital (Jiangxi Zhonghong Boyuan Biotechnology Co., Ltd., Nanchang, China) in normal saline (60 mg/kg), and then infected with 15 ± 2 cercariae through the shaved abdominal skin for 20 min. Nine weeks later, all animals were euthanized by CO_2_ inhalation, and liver tissues were collected for further analysis. This study was approved by the Experimental Animal Ethics Committee of Jiangxi Zhonghong Boyuan Biotechnology Co., Ltd. (Nanchang, China) (No.: LL202403310005). All methods were performed in accordance with applicable ethical guidelines and regulatory requirements.

### 2.9. Histopathology

After euthanasia, liver tissues were collected and fixed with 4% paraformaldehyde (Solarbio, Beijing, China). Liver tissues were paraffin-embedded and stained with hematoxylin and eosin (H&E) as well as Sirius Red (Solarbio, Beijing, China) following standard protocols. Pathological changes in each section were examined using an upright microscope (Olympus, Tokyo, Japan), and quantitative measurements were performed using Image-Pro Plus 6.0 software (Media Cybernetics, Rockville, MD, USA).

### 2.10. RT-qPCR Analysis

To evaluate the expression levels of 7 hub genes (*Lcn2*, *Timp1*, *Cth*, *Cp*, *Hmox1*, *Cbs*, and *Gclc*), total RNA was extracted from mouse liver tissues using TRIzol reagent (CW0580S, CWBIO, Taizhou, China) according to the manufacturer’s instructions. The quality and quantity of RNA were assessed using a NanoPhotometer NP80 ultraviolet spectrophotometer (Implen, Munich, Germany). Total RNA was reverse-transcribed into complementary DNA (cDNA) using the HiScript II Q RT SuperMix for qPCR kit (R233, Vazyme, Nanjing, China). RT-qPCR was performed on a StepOnePlus Real-Time PCR System (Applied Biosystems, Waltham, MA, USA) using ChamQ Universal SYBR qPCR Master Mix (Q711, Vazyme, Nanjing, China). The experimental protocol included initial denaturation at 95 °C for 3 min, followed by 40 cycles of 95 °C for 10 s and 60 °C for 30 s, with *Gapdh* serving as the internal reference gene for data normalization. Melting curve analysis was conducted immediately following the final PCR cycle to confirm primer specificity. The temperature was gradually increased from 60 °C to 95 °C with continuous fluorescence monitoring to detect any non-specific amplification. Primers were synthesized by Shanghai Sangon Biotech Co., Ltd. (Shanghai, China). All qPCR reactions were performed in triplicate for each sample, and the expression levels of target genes were quantified using the 2^−ΔΔCt^ method [30]. The primer sequences used for RT-qPCR are detailed in Supplementary Table S3.

### 2.11. Statistical Analysis

Statistical analysis was performed using R software (version 4.2.2), and data from animal experiment verification were analyzed using GraphPad Prism 8.0 (GraphPad Software, San Diego, CA, USA). Data normality was assessed using the Shapiro–Wilk test. For comparisons between two groups, the independent Student’s *t*-test was applied to normally distributed data with homogeneity of variance. For data that did not meet these assumptions or had a small sample size where normality could not be reliably determined, the non-parametric Mann–Whitney U test was utilized. The Kruskal–Wallis test was used for comparisons involving three or more non-normally distributed groups. Spearman correlation analysis was employed to determine the correlation coefficient between various molecular entities. Data from animal experiment verification were expressed as mean ± standard deviation (SD). Unless otherwise stated, all statistical *p* values were two-tailed, and significance was defined as *p* < 0.05 (*), *p* < 0.01 (**), or *p* < 0.001 (***).

## 3. Results

### 3.1. Dataset Correction

**Figure 1:**
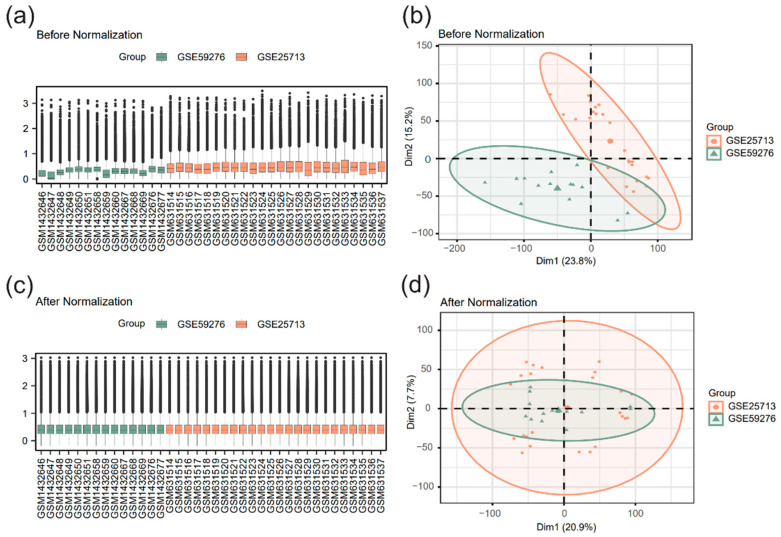
Dataset correction (**a**) Boxplot before correction. (**b**) Boxplot after correction. (**c**) Principal component analysis (PCA) plot before correction. (**d**) PCA plot after correction. Light green represents dataset GSE59276, and light orange represents dataset GSE25713.

To address the heterogeneity inherent in the two datasets (different microarray platforms, mouse strains, and infection duration), R packages sva and limma were utilized to perform batch effect correction and standardization on the merged dataset, thereby ensuring the reliability of the subsequent analysis. Boxplots and principal component analysis (PCA) plots were used to compare the datasets before and after batch effect correction and standardization (Figure 1). The results demonstrated that technical biases and non-biological variations caused by batch effects were significantly mitigated.

### 3.2. DEGs Analysis

**Figure 2:**
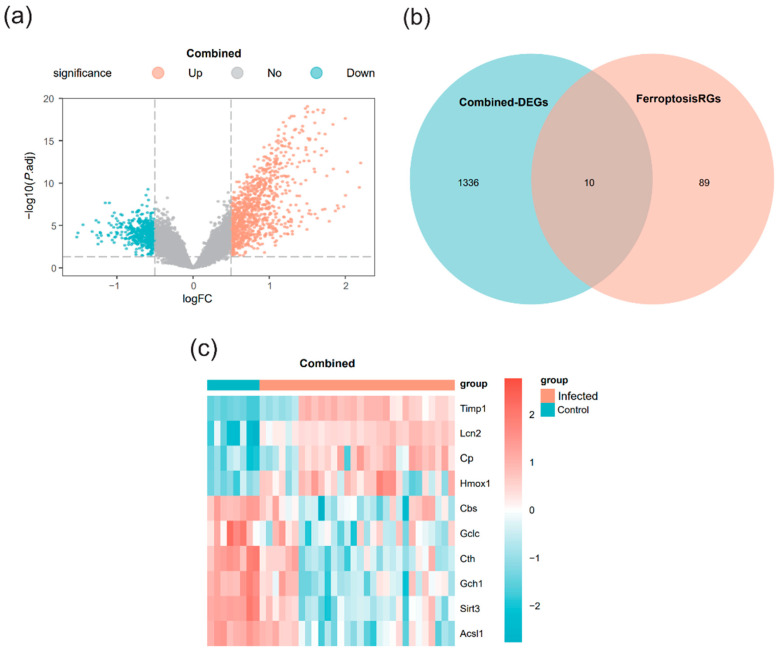
Differentially expressed genes (DEGs) analysis (**a**) Volcano plot of differential analysis results between the infected group and the control group in the merged dataset. (**b**) Venn diagram of DEGs and ferroptosis-related genes (FRGs) in the merged dataset. (**c**) Heatmap of differential expression of ferroptosis-related differentially expressed genes (FRDEGs) in the merged dataset. Light green represents the control group (Control), and light orange represents the infected group (Infected). In the heatmap, red indicates high expression and blue indicates low expression.

The R package limma was used to comprehensively analyze the integrated expression profile data to identify DEGs between the infected and control groups. The analysis identified 1346 genes in the merged dataset meeting the criteria of |logFC| > 0.5 and *p*.adj < 0.05. Specifically, 916 genes were upregulated, and 430 genes were downregulated. A volcano plot visualized these differential analysis results (Figure 2a).

FRDEGs were identified by intersecting the DEGs from the merged dataset with the known FRGs. This process yielded 10 genes (*Lcn2*, *Timp1*, *Sirt3*, *Cth*, *Cp*, *Gch1*, *Acsl1*, *Hmox1*, *Cbs*, and *Gclc*), which were designated as FRDEGs for subsequent analysis. A Venn diagram illustrates this intersection (Figure 2b). A heatmap was generated to depict the differential expression patterns of these 10 FRDEGs, with genes sorted in descending order of their logFC values (Figure 2c).

### 3.3. Functional Enrichment Analysis

We performed GO functional enrichment analysis on the 10 FRDEGs to explore their related BP, MF, and CC (Supplementary Table S4). A term was considered significantly enriched if it met the criteria of *p* < 0.05 and FDR < 0.25. The analysis revealed that these FRDEGs were primarily enriched in biological processes such as sulfur compound biosynthetic and metabolic processes, response to oxidative stress, cysteine metabolic process, and sulfur-containing amino acid metabolic process.

In terms of MF, the analysis identified key terms including Pyridoxal Phosphate Binding, Vitamin B6 Binding, Carbon-Nitrogen (Non-Peptide) Bond Hydrolase Activity, and Heme Binding. Notably, the terms Vitamin B6 Binding and Pyridoxal Phosphate Binding actually point to the coenzyme dependency of the same class of enzymes, as pyridoxal 5′-phosphate (PLP) is the biologically active form of vitamin B6. This co-enrichment feature reflects the specific metabolic functions of the screened hub genes: *Cth* (encoding cystathionine γ-lyase) and *Cbs* (encoding cystathionine β-synthase) are both PLP-dependent rate-limiting enzymes that catalyze the conversion of homocysteine to cysteine in the transsulfuration pathway, acting as central nodes for maintaining intracellular cysteine homeostasis and glutathione synthesis capacity. Simultaneously, *Gclc* (encoding glutamate–cysteine ligase catalytic subunit) functions as the catalytic subunit of the rate-limiting enzyme for glutathione synthesis, directly determining GSH production efficiency; *Cp* (encoding ceruloplasmin) and *Hmox1* (encoding heme oxygenase 1) jointly participate in iron homeostasis and oxidative stress regulation. Specifically, the former exerts ferroxidase activity to facilitate iron efflux, and the latter catalyzes heme degradation to release free iron. In comparison, *Lcn2* (encoding lipocalin 2) and *Timp1* (encoding tissue inhibitor of metalloproteinases 1) are closely associated with the amplification of inflammatory responses and extracellular matrix remodeling, respectively.

**Figure 3:**
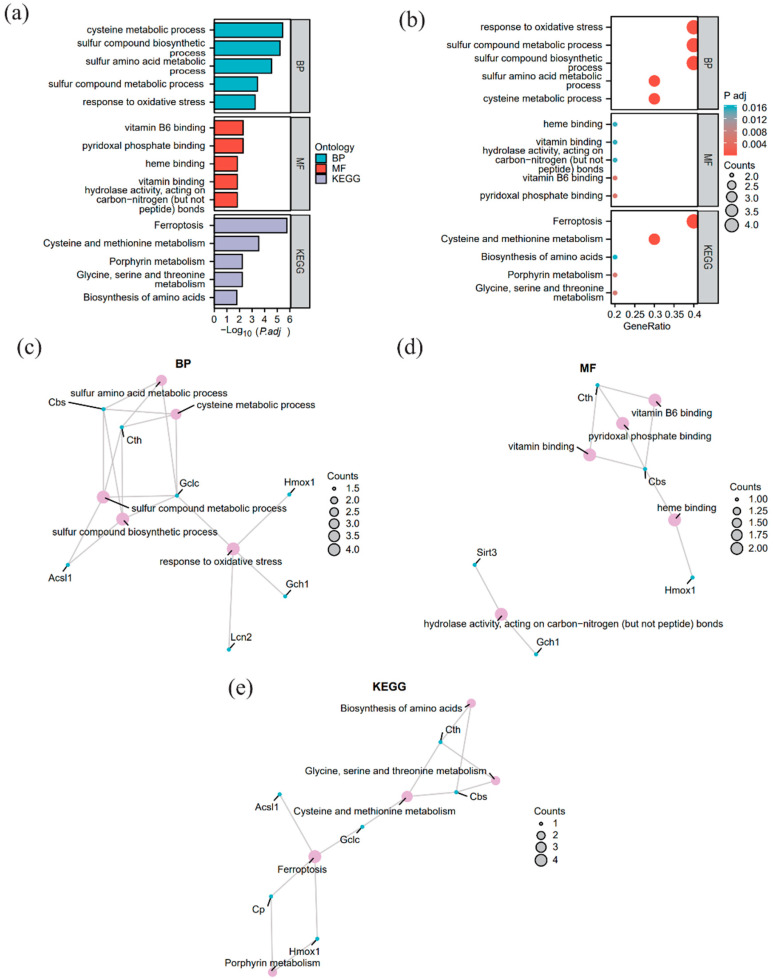
Gene Ontology (GO) and Kyoto Encyclopedia of Genes and Genomes (KEGG) enrichment analysis of FRDEGs (**a**) Comprehensive bar chart (GO biological process [BP], GO molecular function [MF], KEGG). (**b**) Comprehensive bubble chart (GO BP, GO MF, KEGG). (**c**) BP network diagram. (**d**) MF network diagram. (**e**) KEGG pathway network diagram. In the network diagrams (**c**–**e**), pink dots represent specific enriched terms (GO BP, GO MF) or pathways (KEGG), and blue dots represent specific genes. Enriched terms and pathways were filtered using the criteria of *p* < 0.05 and false discovery rate (FDR) < 0.25.

The results are visualized in a bar chart (Figure 3a) and a bubble chart (Figure 3b). Additionally, network diagrams were constructed for BP (Figure 3c), MF (Figure 3d), and KEGG pathways (Figure 3e), depicting the relationships between molecules and their annotations.

### 3.4. GSEA

**Figure 4:**
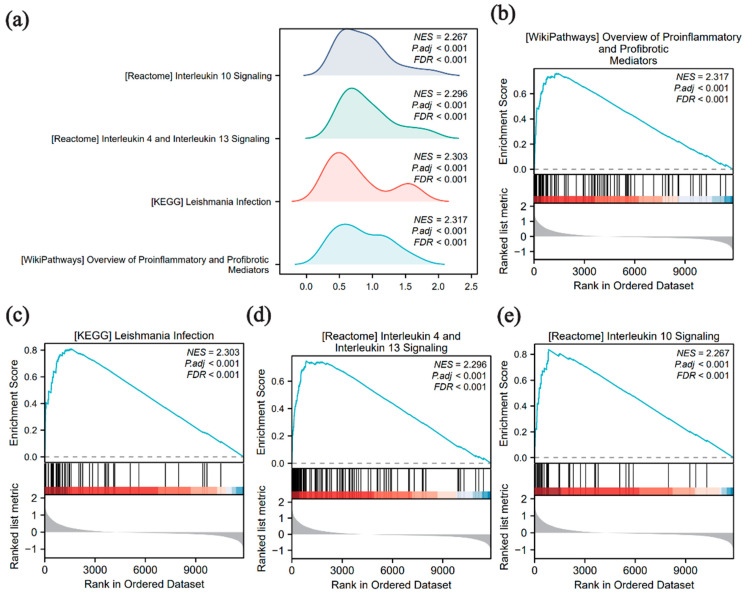
Gene Set Enrichment Analysis (GSEA) enrichment analysis of the merged dataset (**a**) GSEA enrichment ridge plot of the top four biological terms in the merged dataset. (**b**–**e**) Genes in the merged dataset are significantly enriched in pro-inflammatory and profibrotic mediators (**b**), *Leishmania* infection (**c**), interleukin (IL) -4 and IL-13 signaling (**d**), and IL-10 signaling (**e**). The color bar at the bottom represents the ranked list metric, where red indicates genes that are positively correlated with the phenotype (upregulated), and blue indicates genes that are negatively correlated (downregulated). The screening criteria for GSEA were *p*-value (*p*.adj) < 0.05 and FDR < 0.25. *p* value correction was performed using the Benjamini–Hochberg (BH) method.

GSEA was employed to examine the relationship between global gene expression levels and BP, MF, and CC in the merged dataset to better understand their impact on schistosomiasis pathology. Significant pathways were identified using *p*.adj < 0.05 and BH-corrected FDR < 0.25. The four pathways with the highest normalized enrichment scores (NESs) were selected for visualization (Figure 4a–e). The analysis revealed that gene sets in the infected group were significantly enriched in pro-inflammatory and profibrotic mediators, as well as *Leishmania* infection-related pathways (Figure 4b,c). Other enriched pathways included the interleukin (IL)-4/IL-13 signaling pathway (Figure 4d) and the IL-10 signaling pathway (Figure 4e). Additional pathways are listed in Supplementary Table S5.

### 3.5. Differential Expression and ROC Analysis of FRDEGs

**Figure 5:**
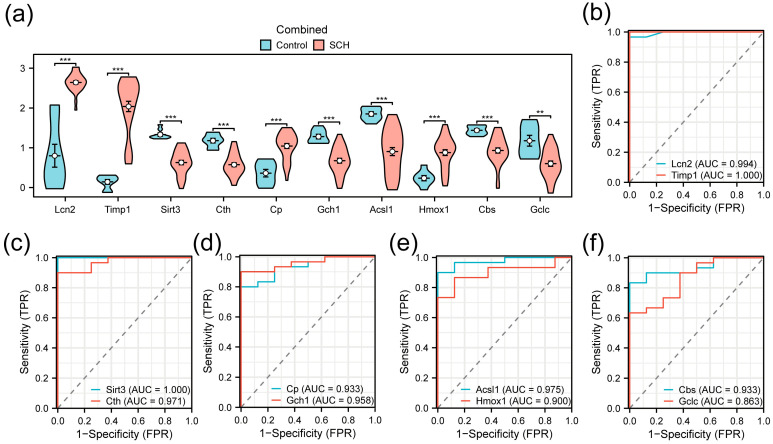
Differential expression analysis and Receiver operating characteristic (ROC) analysis (**a**) Comparison plot of FRDEGs between the infected group and the control group in the merged dataset; (**b**–**f**) ROC curves of FRDEGs: *Lcn2* and *Timp1* (**b**); *Sirt3* and *Cth* (**c**); *Cp* and *Gch1* (**d**); *Acsl1* and *Hmox1* (**e**); *Cbs* and *Gclc* (**f**). ** *p* < 0.01, *** *p* < 0.001.

The Wilcoxon rank-sum test was used to compare the expression levels of the 10 FRDEGs between the infected and control groups. A violin plot visualized these comparisons (Figure 5a). The results showed that all 10 FRDEGs exhibited statistically significant differences. Specifically, *Gclc* showed a significant difference (*p* < 0.01), while the other nine FRDEGs displayed highly significant differences (*p* < 0.001).

ROC curves were generated to evaluate the diagnostic value of these genes (Figure 5b–f). The results indicated that *Lcn2* (AUC = 0.994), *Timp1* (AUC = 1.000), *Sirt3* (AUC = 1.000), *Cth* (AUC = 0.971), *Cp* (AUC = 0.933), *Gch1* (AUC = 0.958), *Acsl1* (AUC = 0.975), *Hmox1* (AUC = 0.900), and *Cbs* (AUC = 0.933) demonstrated high diagnostic accuracy, while *Gclc* (AUC = 0.863) showed moderate diagnostic accuracy.

### 3.6. ImmuCC for Immune Infiltration Analysis

**Figure 6:**
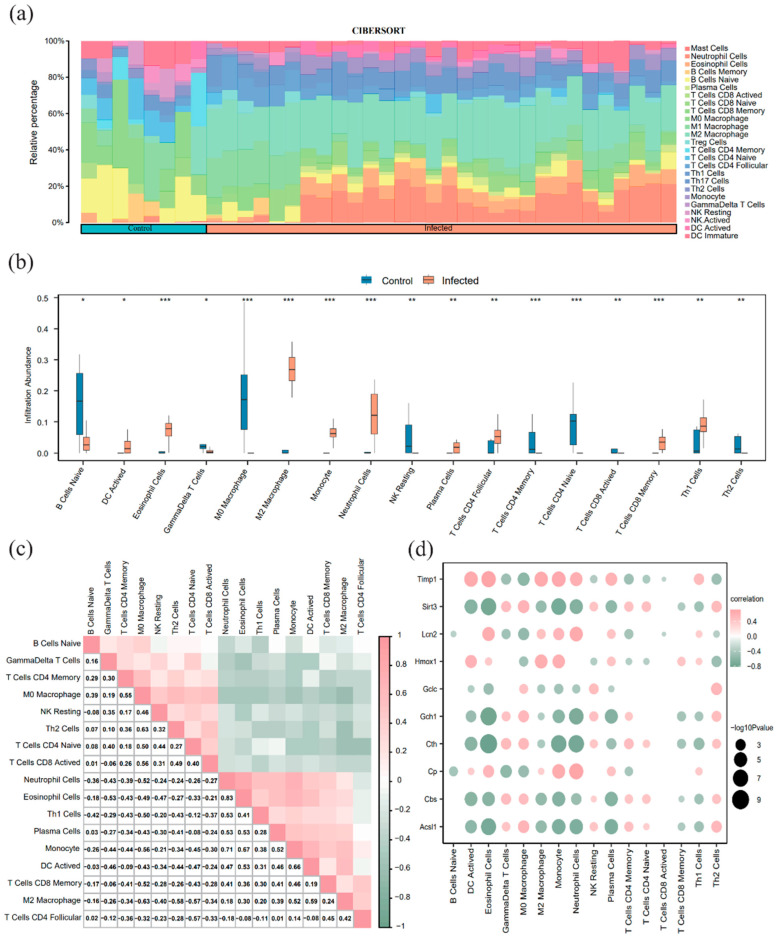
Immune infiltration analysis based on ImmuCC (**a**) Bar chart of immune cell proportions in the merged dataset. (**b**) Difference comparison plot of 17 immune cells between the infected group and the control group. (**c**) Correlation heatmap of immune cell infiltration abundance in the merged dataset.; (**d**) Correlation bubble chart of FRDEGs and immune cell infiltration abundance in the integrated schistosomiasis dataset. A correlation coefficient (*r* value) between 0.3 and 0.5 indicates a weak correlation, and between 0.5 and 0.8 indicates a moderate correlation. Light green indicates a negative correlation; light orange indicates a positive correlation. The color intensity reflects the strength of the correlation. * *p* < 0.05, ** *p* < 0.01, *** *p* < 0.001.

Using the ImmuCC method, we quantified the relative abundance of 25 immune cell types in the merged dataset (Figure 6a). The Wilcoxon rank-sum test identified significant differences in 17 cell types between the infected and control groups (Figure 6b). A correlation heatmap visualized the interactions among these 17 immune cell types (Figure 6c); notably, eosinophils and neutrophils showed the strongest positive correlation (*r* = 0.83), while M2 and M0 macrophages exhibited a significant negative correlation (*r* = −0.63).A correlation bubble chart further illustrated the relationship between the 10 FRDEGs and infiltrating immune cells (Figure 6d). Among the ferroptosis-related genes, *Timp1*, *Lcn2*, and *Cp* were significantly positively correlated with neutrophil abundance (*r* > 0, *p* < 0.05), whereas *Sirt3*, *Gch1*, *Cth*, and *Acsl1* were significantly negatively correlated with dendritic cell (DC) activation (*r* < 0, *p* < 0.05).

### 3.7. PPI Network and Functional Similarity Analysis

**Figure 7:**
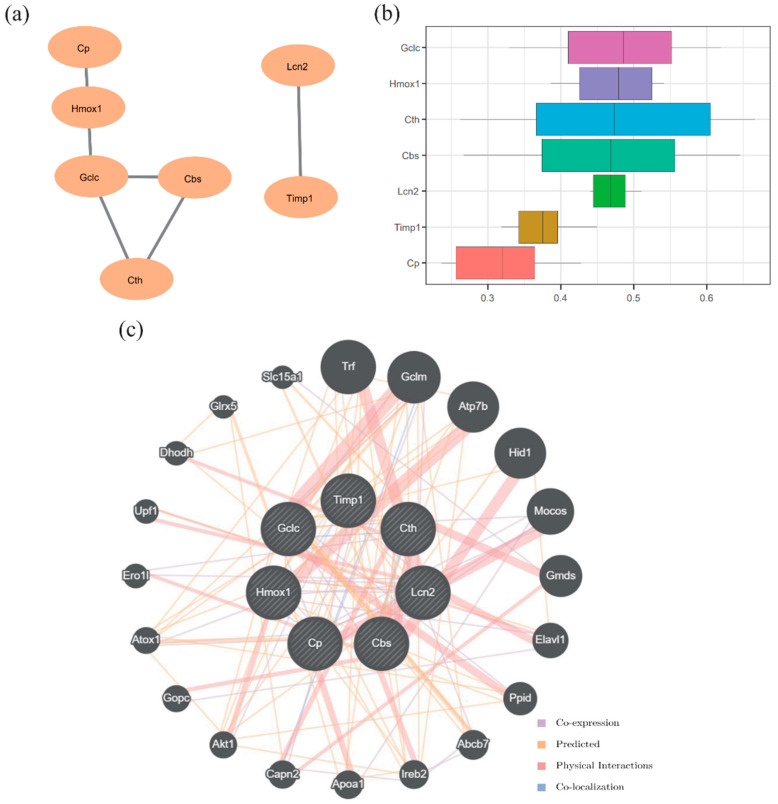
Protein–protein interaction (PPI) network and functional similarity analysis (**a**) PPI network. (**b**) Boxplot of functional similarity of hub genes. (**c**) Functional similarity gene interaction network predicted by GeneMANIA. The circles represent hub genes and their functionally similar partners, and the color of the lines indicates the type of functional connection.

PPI analysis of the 10 FRDEGs was performed using the STRING database (minimum interaction score = 0.700). Genes with valid connections were retained, identifying 7 hub genes (*Lcn2*, *Timp1*, *Cth*, *Cp*, *Hmox1*, *Cbs*, and *Gclc*). The PPI network was visualized using Cytoscape (Figure 7a). Functional similarity analysis using the GOSemSim package revealed that *Gclc* exhibited the highest functional similarity score among the hub genes (Figure 7b). Additionally, GeneMANIA analysis indicated that these 7 hub genes shared co-expression, predicted interactions, physical interactions, and co-localization networks with other genes (Figure 7c).

### 3.8. Histopathological Morphology of Liver Tissues

**Figure 8:**
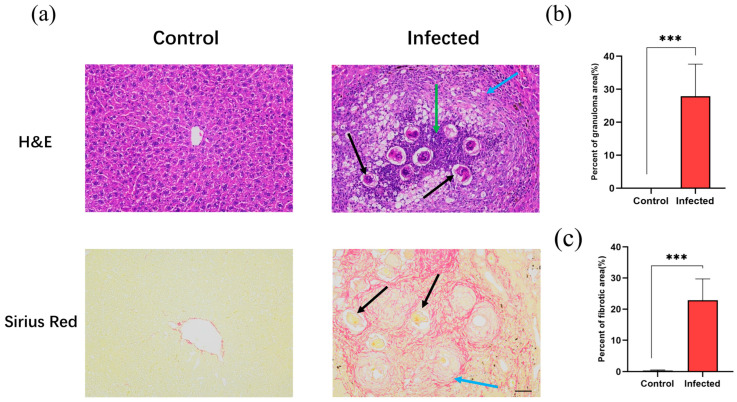
Histopathological changes in liver tissues of the control and infected groups. (**a**) Representative images of hematoxylin and eosin (H&E) and Sirius Red staining from the control and infected groups (magnification, ×200; scale bar, 50 μm). Black arrows indicate *Schistosoma japonicum* Katsurada, 1904 (*S. japonicum*) eggs; green arrows indicate inflammatory cell infiltration; blue arrows indicate fibrotic areas and collagen deposition. (**b**) Quantification of the percentage of granuloma area to the total area based on H&E staining. (**c**) Quantification of the percentage of collagen area to the total area based on Sirius Red staining. *** *p* < 0.001.

To verify the expression patterns of the bioinformatics-screened hub genes in an in vivo system, we established a *S. japonicum*-infected mouse model. H&E and Sirius Red staining were used to evaluate histopathology (Figure 8). In the control group, hepatocytes were arranged neatly with intact lobular structures. In contrast, the infected group exhibited significant structural damage, characterized by pronounced egg granulomas and collagen fiber deposition. While the control group showed no obvious inflammation or necrosis, the infected group displayed extensive inflammatory cell infiltration and local necrosis near granulomas. Sirius Red staining confirmed that the area of collagen fibrosis in the infected group was significantly increased compared to the control group. These results confirmed that the model successfully simulated the core pathological characteristics of schistosomal liver fibrosis.

### 3.9. RT-qPCR Verification of Hub Genes

**Figure 9:**
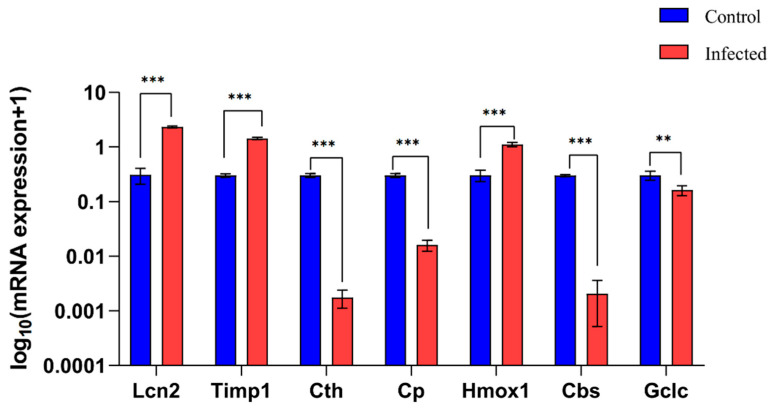
Reverse transcription-quantitative polymerase chain reaction (RT-qPCR) verification results of hub gene expression differences between the control group and the infected group. Data are expressed as mean ± standard deviation (SD) (*n* = 4). ** *p* < 0.01, *** *p* < 0.001.

Following histological confirmation, we validated the mRNA expression levels of the 7 hub genes (*Lcn2*, *Timp1*, *Cth*, *Cp*, *Hmox1*, *Cbs*, and *Gclc*) via RT-qPCR. Consistent with bioinformatics predictions, *Lcn2*, *Timp1*, and *Hmox1* were significantly upregulated, while *Cth*, *Cbs*, and *Gclc* were significantly downregulated in the infected group compared to controls (Figure 9). Only the expression of *Cp* (downregulated in RT-qPCR) was inconsistent with the bioinformatics prediction (upregulation). The consistent validation of 6 out of 7 genes strongly supports their association with schistosomal liver fibrosis, laying a solid foundation for future functional experiments.

## 4. Discussion

In this study, 99 FRGs were utilized as a function-oriented molecular screening set. By integrating the GEO datasets GSE25713 and GSE59276, robust DEGs were identified at the broad level of infected state versus healthy state, minimizing confounding factors arising from heterogeneous clinical or experimental variables (such as specific infection time points, gender, or intervention measures). Combined with multi-dimensional bioinformatics analysis and animal experiment verification, the potential association between FRGs and *S. japonicum*-induced liver fibrosis was systematically explored. Following data merging, strict batch effect correction was performed. The PCA plot showed that samples from different datasets were well integrated after correction, indicating that technical biases were effectively controlled, establishing a robust foundation for subsequent differential analysis. This strategy of dataset merging followed by batch effect correction is a widely recognized method in bioinformatics to enhance statistical power and discover robust hub genes [31,32].

In addition, for exploratory studies, adopting an evidence-based and relatively loose screening strategy, setting the thresholds at |logFC| > 0.5 [33,34], while controlling FDR to below 0.25 [35,36,37], is a common and effective method in the field. To mitigate the risk of false positives associated with a looser FDR, this study employed a multi-dimensional verification approach. The hub genes screened by subsequent PPI network analysis all possess clear protein–protein interaction associations. Immune infiltration analysis showed that they are significantly related to key pathological features of the disease (eosinophil/neutrophil infiltration), and animal experiments verified that the expression trends of 6 of the 7 hub genes are consistent with bioinformatics predictions. This combinatorial strategy of broad screening and rigorous verification not only promotes the comprehensiveness of exploratory research but also supports the credibility of core candidate genes, aligning with established research frameworks in the field [38,39].

A total of 1346 schistosomiasis-related DEGs were identified in this study. Through intersection analysis with the FRGs set, 10 FRDEGs were further screened. The core value of this result lies in proposing a potential molecular link between *S. japonicum* infection and the ferroptosis pathway, providing key candidate targets for exploring the potential interaction mechanisms between the two. Among these 10 FRDEGs, we observed that *Timp1* and *Sirt3* exhibited excellent diagnostic accuracy (AUC = 1.000). *Timp1*, identified as a hub gene in our subsequent analysis, has been previously reported to be highly expressed in schistosomiasis patients, offering high diagnostic specificity [40] and potential for assessing the risk of liver fibrosis progression [41]. Although *Sirt3* was not classified as a hub gene based on network connectivity, its high diagnostic performance warrants attention. As a nicotinamide adenine dinucleotide (NAD^+^)-dependent deacetylase in mitochondria, *Sirt3* participates in regulating HSC activation by modulating mitochondrial protein acetylation and represents a potential therapeutic target for fibrosis intervention [42]. These conclusions highlight the clinical translational value of the FRDEGs screened in this study. While an AUC of 1.000 suggests excellent discrimination, we acknowledge that this perfect separation is likely influenced by the relatively small sample size and the clear-cut distinction between the experimental groups. Therefore, these values should be interpreted as indicators of high potential rather than absolute diagnostic certainty in clinical populations.

Seven hub genes (*Lcn2*, *Timp1*, *Cth*, *Cp*, *Hmox1*, *Cbs*, and *Gclc*) closely related to the pathology of liver fibrosis were identified through the PPI network. Verification by RT-qPCR in a mouse model showed that the ferroptosis marker *Timp1* and drivers *Lcn2* and *Hmox1* were significantly upregulated in the infected group, while ferroptosis inhibitors *Cth*, *Cbs*, and *Gclc* were significantly downregulated, consistent with bioinformatics predictions. Notably, only the downregulation trend of *Cp* was discordant with bioinformatics predictions. This discrepancy suggests that *Cp* expression may be modulated by post-transcriptional regulatory mechanisms; it also highlights the complexity of host responses, as transcriptomic sequencing alone may not fully capture the entire landscape of gene regulation. The overall expression pattern is highly consistent with the molecular characteristics of ferroptosis pathway activation, suggesting that hub genes such as *Lcn2* and *Timp1* may contribute to the occurrence and development of schistosomal liver fibrosis by potentially regulating ferroptosis. Previous studies have indicated that *Lcn2* can promote HSC activation [43], *Timp1* serves as an indicator for fibrosis risk [40], and *Hmox1* is involved in the liver inflammatory response by regulating iron metabolism [44], all of which align with our findings.

Functional enrichment analysis revealed that the FRDEGs were significantly enriched in sulfur compound biosynthesis and metabolic pathways, particularly cysteine and GSH metabolism (Figure 3). This finding provides key clues for unraveling the ferroptosis-related molecular events underlying schistosomal liver fibrosis. Notably, the enrichment of these pathways was primarily driven by three key genes consistently downregulated in the livers of infected mice: *Cth*, *Cbs*, and *Gclc*. It is well established that *Cth* and *Cbs* encode key enzymes catalyzing the production of cysteine, the rate-limiting substrate for GSH synthesis [7], while *Gclc* encodes the critical rate-limiting enzyme for GSH synthesis itself [45]. GSH serves as the essential reducing equivalent for glutathione peroxidase 4 (GPX4), a core inhibitor of ferroptosis [7]. Therefore, the synchronous downregulation of *Cth*, *Cbs*, and *Gclc* suggests that, in the context of *S. japonicum* infection, hepatocytes may undergo a pathogenic process characterized by cysteine deprivation, subsequent GSH depletion, and impaired GPX4 function. This cascade likely reduces the cellular capacity to resist lipid peroxidation, potentially rendering hepatocytes more susceptible to ferroptosis.

Collectively, *S. japonicum* infection appears to compromise the hepatic antioxidant defense system while concurrently activating pro-ferroptotic factors. The downregulation of sulfur metabolism-related genes suggests diminished GSH synthesis capacity, which, combined with dysregulated iron metabolism, may intensify lipid peroxidation stress, providing a potential molecular basis for hepatocyte injury. In parallel, GSEA and immune infiltration analyses revealed significant activation of immune pathways [46,47,48] and marked infiltration of eosinophils and neutrophils. The positive correlation between these infiltrates and *Timp1*/*Lcn2* expression suggests a potential synergy between metabolic imbalance and immune remodeling in driving fibrosis. These findings offer a comprehensive perspective on the formation of a ferroptosis-susceptible state in schistosomiasis-induced liver fibrosis, bridging both metabolic and immune dimensions.

Despite the promising findings, several limitations in this study should be acknowledged. First, the bioinformatics analysis relied on public datasets with relatively limited sample sizes (GSE25713 and GSE59276). Although rigorous batch effect correction was applied to mitigate technical biases, the inherent variability of small-sample datasets may influence the generalizability of the results, warranting further validation in larger, multi-center cohorts. Second, the diagnostic and therapeutic potential of the identified hub genes was primarily verified in a murine model. While animal models provide a controlled environment to study pathological mechanisms, they cannot fully replicate the heterogeneity of human clinical presentations. Future studies involving clinical patient samples stratified by different fibrosis stages are essential to confirm the translational applicability of these findings. Third, our screening of FRGs utilized the GeneCards and MSigDB databases to ensure a broad coverage of high-confidence targets. While this strategy successfully identified core drivers, future investigations incorporating specialized databases, such as FerrDb [49], could offer a more granular analysis of specific ferroptosis sub-pathways. Finally, this study focused on exploring the transcriptomic landscape linking *S. japonicum* infection to ferroptosis. We verified the dysregulation of key hub genes (e.g., downregulation of antioxidant defense genes and upregulation of profibrotic genes), which suggests the activation of the ferroptosis pathway. However, direct biochemical indicators of ferroptosis, such as lipid peroxide accumulation or GPX4 enzymatic activity, were not assessed in this exploratory phase. These functional validations represent a critical next step, for which the current study provides a valuable molecular rationale and targeted direction.

## 5. Conclusions

In this study, we have systematically characterized the ferroptosis-related molecular landscape of *S. japonicum*-induced liver fibrosis, highlighting a potential pathogenic link between suppressed sulfur metabolism and immune microenvironment remodeling. Our findings suggest a dual-hit mechanism potentially driving fibrogenesis: the synchronous downregulation of the *Cth*-*Cbs*-*Gclc* axis likely impairs the hepatic antioxidant defense by limiting cysteine and GSH synthesis, thereby rendering hepatocytes more susceptible to ferroptosis. Concurrently, the upregulation of *Timp1*, *Lcn2*, and *Hmox1* may facilitate a pro-fibrotic and inflammatory immune microenvironment.

Collectively, this study goes beyond identifying a signature of the 7 hub genes to propose a novel metabolic–immune framework for understanding schistosomal liver fibrosis. These hub genes, particularly *Timp1*, offer promising diagnostic potential, while the identified metabolic vulnerabilities highlight the restoration of sulfur metabolism as a potential therapeutic strategy to mitigate ferroptosis and fibrotic progression. Further validation of these targets in clinical cohorts and broad-spectrum infection models is warranted to facilitate their translation into clinical practice.

## Data Availability

Publicly available datasets were analyzed in this study. These data can be found here: Gene Expression Omnibus (GEO) under accession numbers GSE25713 (https://www.ncbi.nlm.nih.gov/geo/query/acc.cgi?acc=GSE25713, accessed on 4 June 2024) and GSE59276 (https://www.ncbi.nlm.nih.gov/geo/query/acc.cgi?acc=GSE59276, accessed on 4 June 2024).

## Supplementary Materials

The following supporting information can be downloaded at: https://www.mdpi.com/article/10.3390/pathogens15020126/s1, Table S1: GEO dataset information list; Table S2: Ferroptosis-related genes list; Table S3: Primers used for reverse transcription-quantitative polymerase chain reaction; Table S4: Results from the GO and KEGG enrichment analyses; Table S5: GSEA enrichment analysis results.

## Abbreviations

The following abbreviations are used in this manuscript:

AUC	Area under the curve
BH	Benjamini–Hochberg
BP	Biological process
CC	Cellular component
cDNA	Complementary DNA
DAMPs	Damage-associated molecular patterns
DC	Dendritic cell
DEGs	Differentially expressed genes
FDR	False discovery rate
FRDEGs	Ferroptosis-related differentially expressed genes
FRGs	Ferroptosis-related genes
GEO	Gene Expression Omnibus
GO	Gene Ontology
GSEA	Gene Set Enrichment Analysis
GSH	Glutathione
H&E	Hematoxylin and eosin
HSCs	Hepatic stellate cells
IL	Interleukin
KEGG	Kyoto Encyclopedia of Genes and Genomes
logFC	Log fold change
MF	Molecular function
MSigDB	Molecular Signatures Database
NAD^+^	Nicotinamide adenine dinucleotide
NESs	Normalized enrichment scores
*p*.adj	Adjusted *p*-value
PCA	Principal component analysis
PLP	Pyridoxal 5′-phosphate
PPI	Protein–protein interaction
ROC	Receiver operating characteristic
ROS	Reactive oxygen species
RT-qPCR	Reverse transcription-quantitative polymerase chain reaction
SD	Standard deviation
SEA	Soluble egg antigen
SPF	Specific pathogen-free
